# Quality medicines in maternal health: results of oxytocin, misoprostol, magnesium sulfate and calcium gluconate quality audits

**DOI:** 10.1186/s12884-018-1671-y

**Published:** 2018-01-30

**Authors:** Chimezie Anyakora, Yetunde Oni, Uchenna Ezedinachi, Adebola Adekoya, Ibrahim Ali, Charles Nwachukwu, Charles Esimone, Victor Abiola, Jude Nwokike

**Affiliations:** 1 Promoting the Quality of Medicines Program, USP, Rockville, MD USA; 2 National Agency for Food and Drug Administration and Control, Abuja, Nigeria; 3 Faculty of Pharmacy, Nnamdi Azikiwe University, Awka, Nigeria

## Abstract

**Background:**

The high level of maternal mortality and morbidity as a result of complications due to childbirth is unacceptable. The impact of quality medicines in the management of these complications cannot be overemphasized. Most of those medicines are sensitive to environmental conditions and must be handled properly. In this study, the quality of oxytocin injection, misoprostol tablets, magnesium sulfate, and calcium gluconate injections was assessed across the six geopolitical zones of Nigeria.

**Method:**

Simple, stratified random sampling of health facilities in each of the political zones of Nigeria. Analysis for identification and content of active pharmaceutical ingredient was performed using high-performance liquid chromatography procedures of 159 samples of oxytocin injection and 166 samples of misoprostol tablets. Titrimetric methods were used to analyze 164 samples of magnesium sulfate and 148 samples of calcium gluconate injection. Other tests included sterility, pH measurement, and fill volume.

**Results:**

Samples of these commodities were procured mainly from wholesale and retail pharmacies, where these were readily available, while the federal medical centers reported low availability. Approximately, 74.2% of oxytocin injection samples failed the assay test, with the northeast and southeast zones registering the highest failure rates. Misoprostol tablets recorded a percentage failure of 33.7%. Magnesium sulfate and Calcium gluconate injection samples recorded a failure rate of 6.8% and 2.4%, respectively.

**Conclusion:**

The prevalence of particularly of oxytocin and misoprostol commodities was of substandard quality. Strengthening the supply chain of these important medicines is paramount to ensuring their effectiveness in reducing maternal deaths in Nigeria.

## Background

Complications due to pregnancy are among the leading causes of maternal deaths worldwide. According to a World Health Organization (WHO) 2015 report, approximately 830 women die every day from preventable causes related to pregnancy and childbirth and 99% of these deaths occur in low-resource countries [1]. The global distribution of maternal deaths is influenced by two regions, sub-Saharan Africa and southern Asia, which account for the majority of all maternal deaths [2]. In 2015, two countries, Nigeria and India, accounted for more than one-third of all global maternal deaths, with approximately 58,000 maternal deaths (19%) in Nigeria and 45,000 maternal deaths (15%) in India [3]. Ten countries accounted for nearly 59% of global maternal deaths: in addition to Nigeria and India, they are the Democratic Republic of Congo (22,000), Ethiopia (11,000), Pakistan (9700), Tanzania (8200), Kenya (8000), Indonesia (6400), Uganda (5700), and Bangladesh (5500).

A high percentage of women in these regions deliver at home or outside a health facility without access to obstetric care or a skilled birth attendant. This makes them more vulnerable to complications related to childbirth [4]. Therefore, there is increased interest in finding alternative ways to make low-cost treatments available to improve women’s health in these low-resource countries.

Say et al. reported that, between 2003 and 2009, direct causes were responsible for nearly 72.5% of maternal deaths while indirect causes were responsible for 27.5% of maternal deaths [5]. Postpartum hemorrhage (PPH) is a leading cause of maternal morbidity and mortality [6–8] and accounted for 27.1% of maternal mortality. PPH may be defined as a vaginal loss of blood ≥500 mL immediately after labor or within 24 h after birth [6, 9]. It is usually caused by excessive bleeding due to lack of efficient uterine contraction (uterine atony), vaginal or cervical tears, or tears of the genital system [10–12]. Anemia during pregnancy has been associated with PPH [13, 14]. WHO recognizes injectable oxytocin as the safest and most effective medicine central to the prevention and treatment of PPH [15, 16]. It is currently on the WHO Essential Medicines List [17] and the United Nations Commission on Life-Saving Commodities list of 13 critical commodities [18]. Oxytocin is widely available in low income countries and is fairly inexpensive. Yet challenges in health sector infrastructure and health service delivery can create barriers to oxytocin use [19]. Most countries require that oxytocin injection be administered by trained health care workers, so women delivering at home or with a traditional birth attendant may not have access to oxytocin [16].

In 2013, the Promoting the Quality of Medicines (PQM) program—funded by the U.S. Agency for International Development (USAID) and implemented by United States Pharmacopeia (USP)—conducted a study in Ghana in collaboration with the Ghanaian Food and Drug Authority. The research team found that 55.6% of the 169 oxytocin samples analyzed were found to contain less active pharmaceutical ingredient than label claim. In addition, 39 of 40 samples (97.5%) that were tested also failed the test for sterility [20]. In 2014, an unpublished study carried out in ten regional zones in Ghana showed similar results. In 2016, Torloni et al. conducted a systematic review on the quality of oxytocin in low- and middle-income countries (LMICs), synthesizing studies that contained results of quality tests of 559 samples of oxytocin [21]. The most common reason for failure was an insufficient amount of active pharmaceutical ingredient (API). The 2015 WHO survey of the quality of medicines identified by the United Nations Commission showed a 64.0% failure of oxytocin samples when tested against pharmacopeial specifications, including appearance, identification, assay, related substances, pH measurement, and extractable volume [22].

Besides the challenges with the quality of oxytocin, other factors that hinder the use of oxytocin in low-resource regions include the need for refrigeration, intravenous and intramuscular injection, and lack of trained providers for administration. Oxytocin can be viewed as a tracer medicine for gauging the performance of regulatory systems in LMICs for several reasons, and marketing approval from the local authority provides insight into premarketing and inspection capacity. Oxytocin is time and temperature sensitive; exposure to high temperatures degrades the product. If countries have reliable post-marketing surveillance (PMS) systems, good distribution and storage practices, they can ensure the integrity of the product throughout the supply chain.

Prevention and treatment of PPH in low-resource settings by conventional uterotonics has been challenging, as the desired impact is not being achieved [23–25]. Prostaglandins have been considered an alternative. Misoprostol is a heat-stable prostaglandin E1 analogue used in the prevention and treatment of peptic ulcer disease caused by prostaglandin-synthetase inhibitors. It has been shown to be a potent uterotonic [25] with the ability to increase uterine tone and decrease postpartum bleeding [26]. Misoprostol has been studied as an alternative to oxytocin due to its low cost, stability at room temperature, wide availability, and ease of administration. It can be administered sublingually, orally, vaginally, and rectally [27–29].

The second most common cause of maternal mortality, preeclampsia/eclampsia (PE/E) is a hypertensive, multisystem disorder of pregnancy with no known underlying cause and is usually associated with raised blood pressure and proteinuria [30–32]. In 1992, a global estimate of 50,000 maternal deaths were attributed to eclampsia [33]. In Nigeria, eclampsia is a major cause of obstetric complications, [34, 35] reported to have contributed to 46.3% and 43% of maternal deaths in Kano State [36] and Jigawa State [37], respectively.

Although delivery remains the ultimate treatment for preeclampsia, [38, 39] magnesium sulfate is the drug of choice for preventing eclamptic seizures and also has the additional benefit of reducing the incidence of placental abruption [40, 41]. Occasionally, the administration of magnesium sulfate results in an accidental overdose leading to respiratory paralysis, central nervous system depression and cardiac arrest. Intravenous calcium gluconate, an antidote that quickly reverses the effects of magnesium toxicity, may be required when moderate to severe toxicity occurs [42–44].

With a population greater than 170 million people, Nigeria is a very attractive market for pharmaceuticals and other regulated products. Over 60% of medicines are imported into the country, due to the gap created by inadequate in-country production of essential medicines. With a maternal mortality ratio of 814 per 100,000 live births, it is obvious that Nigeria has a weak health care delivery system, particularly in the area of maternal health. This is exacerbated by the availability of poor-quality medicines, due to either inadequate storage and distribution or poor manufacturing practices. Hence, constant monitoring of the quality of medicines in circulation is a public health necessity. As is the case with most LMICs, quality remains an issue, and the impact of poor-quality medicines cannot be overemphasized. In the case of the uterotonics and drugs for preeclampsia, the implication for poor-quality treatment can prove dire. In this study, quality levels of four maternal commodities in the six geopolitical zones of Nigeria were assessed.

## Methods

### Sampling

To determine sample size, the following criteria were taken into account: population size; confidence level with an assumption of a normal 95% distribution of sample values; and degree of variability in the quality of oxytocin, magnesium sulfate, calcium gluconate injections, and misoprostol tablets distributed across the study sites.

The following factors were carefully considered in drafting the sampling plan:

- Availability of oxytocin, magnesium sulfate, calcium gluconate injections, and misoprostol at the federal medical centers (FMC), state hospitals (SH), primary health care centers (PHCC), and midwife clinic (MWC) levels
- Budgetary and time constraints
- Geographical access
- The laboratory’s standard operating procedures for testing to ensure adequate samples for triplicate testing and handling of out-of-specification occurrences

Table 1 describes distribution of the samples across the different levels of sampling sites. These included FMCs, SHs, PHCCs, MWCs, wholesale pharmacies, retail pharmacies, and patent and proprietary medicine vendors (PPMVs). A total of 234 samples per commodity were determined as an adequate sample size that would represent the true situation in this study. Even though the PPMVs are not permitted to stock these prescription-only commodities, sampling was conducted due to previous records of illegal stocking of ethical pharmaceutical products.

**Table 1:** Sample Distribution

Level of Sampling	No. of Outlets	No. of Samples from Each Outlet	Quantity of Commodity per Sample
Level 1: Patent Medicine Stores	5 patent stores	1 sample of different brands or lots from EACH outlet	Sample A: 10 ampoules each of oxytocin, calcium gluconate, and magnesium sulfate + 20 tablets of misoprostol
Level 2: Wholesale and Retail Pharmacy Stores	4 pharmacies	2 samples of different brands or lots from EACH outlet	Sample A: 30 ampoules each of oxytocin, calcium gluconate and magnesium sulfate + 60 tablets of misoprostol
			Sample B: 30 ampoules each of oxytocin, calcium gluconate and magnesium sulfate + 60 tablets of misoprostol
Level 3: Midwife Clinics	5 MWCs	2 samples of different brands or lots from EACH outlet	Sample A: 10 ampoules each of oxytocin, calcium gluconate and magnesium sulfate + 20 tablets of misoprostol
			Sample B: 10 ampoules each of oxytocin, calcium gluconate and magnesium sulfate + 20 tablets of misoprostol
Level 4: Primary Health Care Centers	5 PHCCs	2 samples of different brands or lots from EACH outlet	Sample A: 10 ampoules each of oxytocin, calcium gluconate and magnesium sulfate + 30 tablets of misoprostol
			Sample B: 10 ampoules each of oxytocin, calcium gluconate and magnesium sulfate + 30 tablets of misoprostol
Level 5: State Hospital Stores	1 SH	4 samples of different brands or lots	Sample A: 15 ampoules each of oxytocin, calcium gluconate and magnesium sulfate + 30 tablets of misoprostol
			Sample B: 15 ampoules each of oxytocin, calcium gluconate and magnesium sulfate + 30 tablets of misoprostol
			Sample C: 15 ampoules each of oxytocin, calcium gluconate and magnesium sulfate + 30 tablets of misoprostol
			Sample D: 15 ampoules each of oxytocin, calcium gluconate and magnesium sulfate + 30 tablets of misoprostol
Level 6: FMC Warehouse	1 FMC	2 samples of different brands or lots	Sample A: 15 ampoules each of oxytocin, calcium gluconate and magnesium sulfate + 30 tablets of misoprostol
			Sample B: 15 ampoules each of oxytocin, calcium gluconate and magnesium sulfate + 30 tablets of misoprostol

Sampling was carried out in six regions across the country during the period May 23–28, 2016: Abia State for the southeast, Edo State for the south-south, Ogun State for the southwest, Kwara State for the north-central, Kaduna State for the northwest, and Bauchi State for the northeast.

At the point of sampling, samples were maintained at the stated manufacturers’ storage conditions while Oxytocin samples were stored in a cold box to ensure the 2 °C–8 °C storage temperature rule was preserved. The temperature of the cold box was constantly monitored and documented until arrival of the products in the laboratory.

### Identification and quantification

Packages of the sampled products were first inspected for correct labelling and packaging according to the requirements of the regulatory authority. These requirements include name of drug product, list of active ingredient, batch number, National Agency for Food and Drug Administration and Control (NAFDAC) number, expiry date, storage conditions, indication for use, name and address of manufacturer, and visibility of particulate matter in injectable.

### Content analysis

Analysis of the API was conducted using the respective compendia methods (USP 39) for oxytocin and USP Pharmacopeial Forum Vol. 26 (5) [Sept–Oct 2000] for misoprostol tablets. Titrimetric method was used for magnesium sulfate and calcium gluconate, as recommended in the current official monographs (BP 2016 and USP 39). Other tests that were carried out to ascertain the quality of these samples included sterility, identification, pH measurement, and fill volume.

High-performance liquid chromatography (HPLC) analyses were conducted on Dionex Ultimate 3000 system and Hitachi Lachrom with Chromeleon and EZChrom software, respectively. Dionex Ultimate 3000 was employed for the analysis of misoprostol, while Hitach Lachrom was employed for oxytocin analysis. The HPLC systems consisted of a quaternary analytical pump, autosampler, and ultraviolet (UV) detector. For misoprostol, the column used was an Ascentis C8 (150 mm × 4.6 mm i.d.; 3 μm particle size); UV detection was performed at 200 nm, while the injection volume was maintained at 100 μL. An online isocratic mobile phase containing a filtered and degassed mixture of methanol, water, and acetonitrile (40:35:25) was employed at a flow rate of 1.5 mL/min and run time of 15 min. The peak responses of the standard and sample solution chromatographs were recorded at a retention time of about 5.50 min.

In the analysis of oxytocin, the column used was C18 (120 mm × 4.6 mm i.d. with a 5 μm particle size). UV detection was performed at 220 nm, and the injection volume was maintained at 100 μL. The system was equilibrated with ratio 7:3 gradient mixtures of mobile phase A (0.1 M monobasic sodium phosphate) and mobile phase B (acetonitrile and water in ratio 1:1) (Table 2). A flow rate of 1.5 mL/min was employed, with a run time of approximately 10 min for oxytocin.

**Table 2:** Gradient Elution for Oxytocin

Time (min)	Mobile phase A (% *v*/v)	Mobile phase B (% v/v)	Comments
0–5	100	0	Isocratic
5–20	70	30	Linear gradient
20–50	30	70	Linear gradient
50–51	60	40	Return to initial composition
51–65	100	0	Re-equilibration

## Results

Samples collected consisted of four commodities from six levels of health facilities (Table 3). The expected number of samples as derived from the sample size calculation could not be achieved because the availability of these products in most of the public health facilities was limited.

**Table 3:** Total Number of Products Sampled from the Health Facilities

Facility	Oxytocin	Misoprostol	Magnesium Sulfate	Calcium Gluconate	Total
Pharmacy	73	79	65	68	285
MWC	46	39	37	28	150
PHCC	37	31	32	31	131
Patent Medicine Store	26	32	22	14	94
SH Store	16	13	16	9	54
FMC	12	8	11	11	42
Total	210	202	183	161	756

In addition, due to damaged samples and incomplete packaging information, the number of analyzed products for each commodity varies with the number of sampled products. It is worthy to note that owing to the relative difference in the number of samples obtained from the different regions, reported failure rates must be interpreted independently. Analysis of the sample data was performed using the R Language and Environment for Statistical Computing.

### Oxytocin

**Fig. 1:**
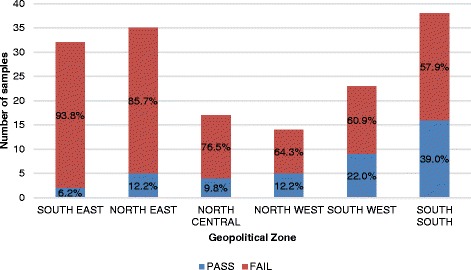
Percentage Failure of Oxytocin Samples by Geopolitical Zone

A total of 159 samples of oxytocin were analyzed and subjected to registration verification. It was observed that all the samples were registered with the national medicines regulatory authority. Labelling information regarding dosage form, brand name, active ingredient, and batch number were provided. Expiry dates of the samples were recorded and found to be within a year at the time of sampling. Although all samples passed visual inspection and complied with the specification from monographs for other parameters tested, the percentage composition of the active ingredient varied between 0.0% and 163.7%. HPLC assay results showed that 74.2% (118/159) did not meet the specification for assay (between 90.0% and 110.0%) as indicated in monograph USP 39. Figure 1 gives a summary of these results categorized by regions of collection.

**Fig. 2:**
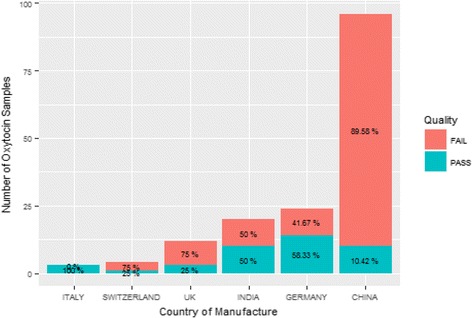
Quality of Oxytocin by Country of Manufacture

According to the package labels, the majority of samples were imported, with 60.4% manufactured in China, 12.6% in India, 15.1% in Germany, and none in Nigeria. Figure 2 shows the percentage of samples from different countries and their failure rates.

**Fig. 3:**
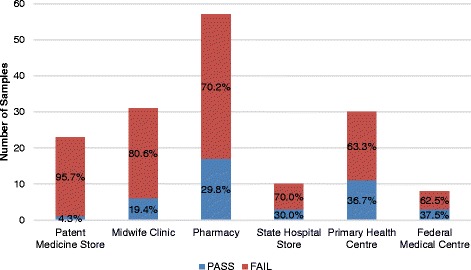
Percentage Failure of Oxytocin per Level of Health Facility

In this study, samples from the patent medicine store were shown to have the highest percentage of failed oxytocin, with a 95.7% (22/23) fail, followed closely by the midwife clinic at 80.6% (25/31), as shown in Fig. 3.

**Fig. 4:**
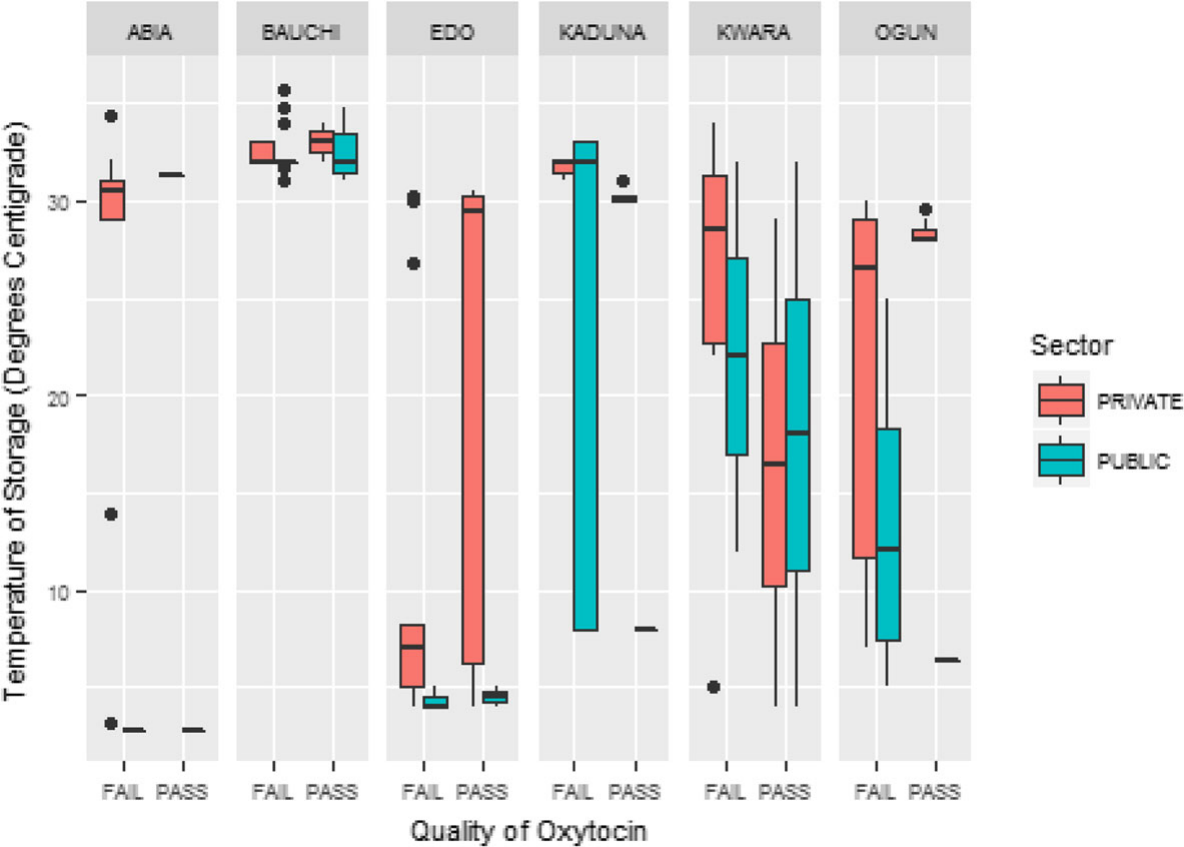
Storage Conditions of Oxytocin and Quality Effect from Each Geopolitical Zone

Due to the effect of higher temperatures on oxytocin injection, storage conditions at the site of sampling were recorded. As shown in the boxplot in Fig. 4, storage temperature ranges for oxytocin samples were observed to be higher in the northern regions, with over 50% of outlets in each collection site of the region recording highs of 30 °C–33 °C and lows of 8 °C–11 °C. Higher storage temperatures ranges were observed in samples from private health facilities compared to those from the public sector in all regions of the geopolitical zones. Although high temperatures were also observed for samples that passed the assay, this was only a small subsection of the samples. Recommended storage temperature range of 2 °C–8 °C was observed in a number of facilities in the southern regions, where a greater number of samples passed the assay test.

### Misoprostol

**Fig. 5:**
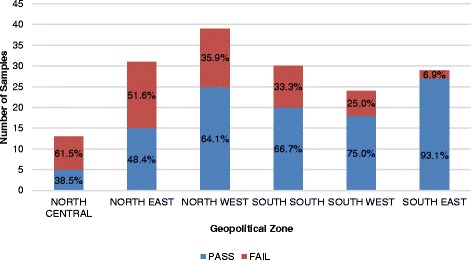
Percentage Failure of Misoprostol by Geopolitical Zone

A total of 166 samples of misoprostol were subjected to registration verification. It was observed that all samples were registered with the national medicines regulatory authority. Labelling information regarding dosage form, brand name, active ingredient, and batch number were provided. Expiry dates of the samples were recorded and found to be within a year at the time of sampling. Although all samples passed visual inspection, the percentage composition of the active ingredient varied grossly. HPLC assay results showed that 33.7% (56/166) did not meet the monograph specification for assay content (between 90.0% and 110.0%). Figure 5 gives a summary of these results categorized by regions of collection.

**Fig. 6:**
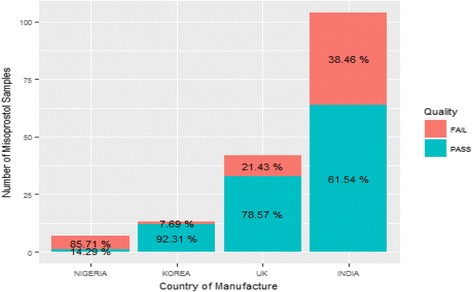
Percentage Failure of Misoprostol by Country of Manufacture

According to the package labels, the majority of the samples were imported, with 62.7% manufactured in India, 25.3% in the United Kingdom, 7.8% in Korea, and 4.2% in Nigeria. Figure 6 shows the percentage of samples from different countries and their failure rates.

**Fig. 7:**
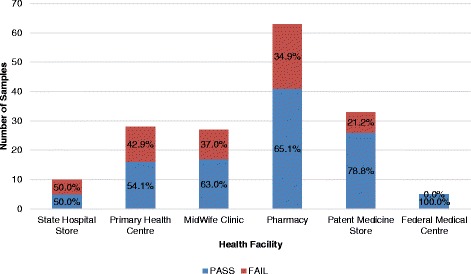
Percentage Failure of Misoprostol Tablets per Level of Health Facility

In this study, samples from the state hospital store were shown to have the highest percentage of failed misoprostol tablets, with a 50.0% (5/10) fail, followed closely by PHCCs with a fail of 42.9% (12/28). The midwife clinics and patent medicine stores recorded a percentage failure of 37.0% (10/27) and 21.2% (7/33), respectively, while the retail and wholesale pharmacy observed a 34.9% (22/63) fail. No failure was recorded from the samples obtained from the FMCs, as shown in Fig. 7.

**Fig. 8:**
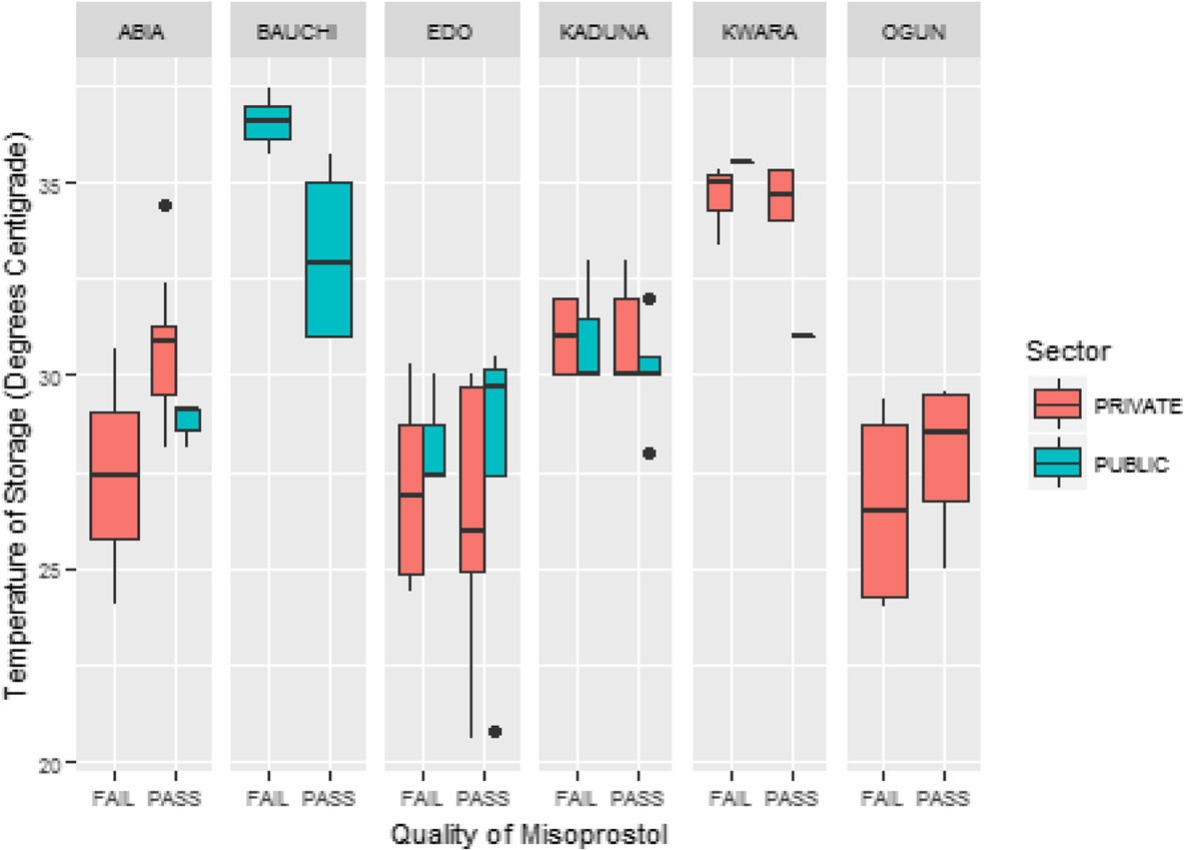
Storage Conditions of Misoprostol Tablets and Quality Effect from Each State

Storage conditions have been shown to play a major role in the quality of misoprostol tablets, with absorption of moisture being the foremost concern. Minimum storage temperature of the products was found in the south-south and southeast regions, at 20.5 °C and 26.0 °C as illustrated in Fig. 8. The boxplots show that storage temperatures of samples procured from the northern region of Kaduna and Kwara were above 30 °C. Although 60% of the samples were sampled from the private sector, there was no distinction between the storage temperatures of the samples from the public and the private sectors.

The quality of misoprostol tablets varied by geopolitical zone, with the northern part recording the highest prevalence of poor-quality misoprostol. However, the southern zones did not perform considerably better, with percentage failures of 33.3% and 25.0% for the south-south and southwest zones, respectively.

### Magnesium sulfate

**Fig. 9:**
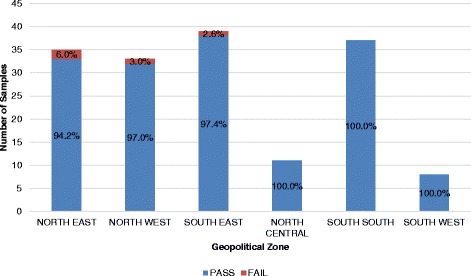
Percentage Failure of Magnesium Sulfate by Geopolitical Zone

A total of 163 samples were subjected to registration, verification and analyzed for the presence of API and sterility. A total of 160 magnesium sulfate samples were shown to have passed the test, while four samples failed to meet the monograph specification range of 93.0% and 107.0% of the labelled amount of magnesium sulfate. Figure 9 gives the distribution of these samples across different geopolitical zones.

### Calcium gluconate

**Fig. 10:**
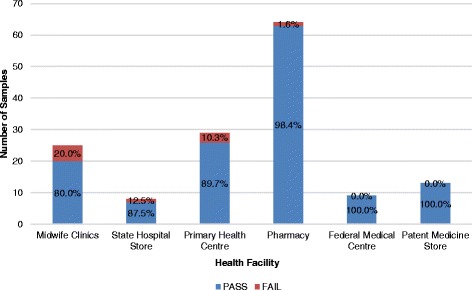
Percentage Failure of Calcium Gluconate by Facility

**Fig. 11:**
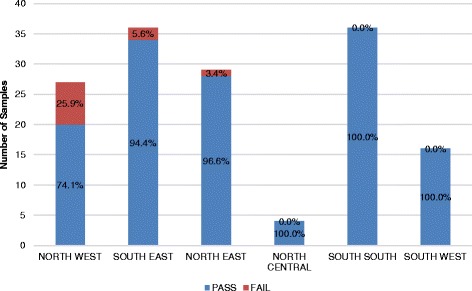
Percentage Failure of Calcium Gluconate by Geopolitical Zone

A total of 148 samples were analyzed for the presence of API and sterility, and 138 samples of calcium gluconate were shown to have passed the test, while 10 samples failed to meet the monograph specification range of 95.0% and 105.0% of the labelled amount of calcium gluconate. Figs. 10 and 11 give the distribution of these samples across different levels of facilities and in different geopolitical zones, respectively.

## Discussion

Injectable oxytocin and misoprostol tablets recorded percentage failures of 74.2% and 33.7%, respectively. In contrast, magnesium sulfate and calcium gluconate injections were both found to be of rather good quality, with 2.4% and 6.8% failure rates, respectively.

The high failure rate of first-line PPH treatment portends a serious health implication and may be a contributing factor in the high maternal mortality ratio in Nigeria. The southeastern and northeastern part of the country recorded the highest failure rate of 25%, while lower failure rates were observed in the south-south and southwest regions. WHO recommends that oxytocin be refrigerated or stored at a temperature of 2 °C–8 °C but low-resourced countries may lack facilities required for proper storage of these products, so storage conditions were equally assessed in the health facilities where sampling was carried out. This result is logical as can be seen from Fig. 4 where the average storage temperature of Oxytocin in the southeastern and northeastern part of Nigeria were above 30 °C unlike the situation in the other regions of the country. Likely causes of this troubling failure rate could be degradation as a result of improper storage as well as poor manufacturing practices.

Misoprostol was observed to have high failure rates in the northeast and south-south regions of the country. Storage conditions of misoprostol tablets though not as critical as the conditions of Oxytocin, is affected by temperature and humidity and is recommended to be stored at a temperature range of 25 °C–30 °C. This recommended temperature was observed in about 50% of the sites, while the others recorded storage temperatures above 30 °C. Furthermore, one of the sampled products was observed to be falsified, as no active ingredient was found.

The northern region of the country reported most of the failure rates of magnesium sulfate, while calcium gluconate injections sampled in the northwest and southeast regions showed relatively poorer quality products. In comparing failure rates across geographic zones and across levels in the supply chain, it is instructive to know that sample sizes were not the same in the various geographic zones and levels; therefore, differences in failure rates should be interpreted with caution. As a result, absolute failure rates are more interpretive than relative failure rates.

### Limitations

A baseline study of the quality of the studied commodities was not established prior to the study. The prevalence of poor-quality medicines may also have been attributed to the initial quality, but this issue was not addressed by our study. In addition, while post-partum hemorrhage is a major cause of maternal deaths, there are no reliable data correlating the prevalence of maternal deaths in Nigeria with the quality of the medicines used in the treatment of post-partum hemorrhage.

## Conclusion

The level of substandard oxytocin injections and misoprostol tablets was significantly high. The storage conditions could be a possible source of this level of failure as both oxytocin injections and misoprostol tablets degrade rapidly if poorly stored. Stronger regulatory action by the Medicines Regulatory Authority (MRA), engagement and education of all key players in the pharmaceutical supply chain could ensure they have a more full understanding of the storage conditions of these commodities. Also, more frequent post-marketing surveillance of these maternal health commodities could help to improve substandard medication given to women in need.

## Abbreviations

API: Active pharmaceutical ingredient
FMC: Federal medical center
HPLC: High-performance liquid chromatography
LMIC: Low- and middle-income country
MCH: Maternal and child health
MWC: Midwife clinic
NAFDAC: National Agency for Food and Drug Administration and Control
PE/E: Preeclampsia/eclampsia
PHCC: Primary health care center
PMS: Post-marketing surveillance
PPH: Postpartum haemorrhage
SH: State hospital
UV: Ultraviolet
WHO: World Health Organization
